# Exploring the utility of ultrasound to assess disuse atrophy in different muscles of the lower leg

**DOI:** 10.1002/jcsm.13583

**Published:** 2024-08-26

**Authors:** Edward J. Hardy, Joseph J. Bass, Thomas B. Inns, Mathew Piasecki, Jessica Piasecki, Craig Sale, Robert H. Morris, Jonathan N. Lund, Ken Smith, Daniel J. Wilkinson, Philip J. Atherton, Bethan E. Phillips

**Affiliations:** ^1^ Centre of Metabolism, Ageing & Physiology (COMAP), MRC‐Versus Arthritis Centre for Musculoskeletal Ageing Research (CMAR), and Nottingham NIHR Biomedical Research Centre University of Nottingham, School of Medicine Derby UK; ^2^ Department of Surgery Royal Derby Hospital Derby UK; ^3^ School of Science and Technology Nottingham Trent University Nottingham UK; ^4^ Institue of Sport Manchester Metropolitan University Manchester UK

**Keywords:** Disuse, Imaging, MRI, Muscle, Ultrasound

## Abstract

**Background:**

Skeletal muscle is a highly plastic tissue crucial for many functions associated with whole‐body health across the life course. Magnetic resonance imaging (MRI) is the current gold standard for measuring skeletal muscle size. However, MRI is expensive, and access to facilities is often limited. B‐mode ultrasonography (U/S) has been proposed as a potential alternative to MRI for the assessment of muscle size. However, to date, no work has explored the utility of U/S to assess disuse muscle atrophy (DMA) across muscles with different atrophy susceptibility profiles, an omission which may limit the clinical application of previous work.

**Methods:**

To address this significant knowledge gap, 10 young men (22 ±  years, 24.1 ± 2.3 kg/m^2^) underwent 15‐day unilateral leg immobilization using a knee‐brace and air boot. Cross‐sectional area (CSA) and muscle thickness (MT) of the tibialis anterior (TA) and medial gastrocnemius (MG) were assessed via U/S before and after immobilization, with CSA and muscle volume assessed via MRI.

**Results:**

With both muscles combined, there were good correlations between each U/S and MRI measure, both before (e.g., CSA_MRI_ vs. MT_U/S_ and CSA_U/S_: *r* = 0.88 and 0.94, respectively, both *P* < 0.0001) and after (e.g., VOL_MRI_ vs. MT_U/S_ and CSA_U/S_: *r* = 0.90 and 0.96, respectively, both *P* < 0.0001) immobilization. The relationship between the methods was notably stronger for MG than TA at each time‐point (e.g., CSA_MRI_ vs. MT_U/S_: MG, *r* = 0.70, *P* = 0.0006; TA, *r* = 0.37, *P* = 0.10). There was no relationship between the degree of DMA determined by the two methods in either muscle (e.g., TA pre‐ vs. post‐immobilization, VOL_MRI_: 136 ± 6 vs. 133 ± 5, *P* = 0.08; CSA_U/S_: 6.05 ± 0.3 vs. 5.92 ± 0.4, *P* = 0.70; relationship between methods: *r* = 0.12, *P* = 0.75).

**Conclusions:**

Both MT_U/S_ and CSA_U/S_ provide comparable static measures of lower leg muscle size compared with MRI, albeit with weaker agreement in TA compared to MG. Although both MT_U/S_ and CSA_U/S_ can discern differences in DMA susceptibility between muscles, neither can reliably assess degree of DMA. Based on the growing recognition of heterogeneous atrophy profiles between muscles, and the topical importance of less commonly studied muscles (i.e., TA for falls prevention in older adults), future research should aim to optimize accessible methods to determine muscle losses across the body.

## Introduction

Skeletal muscle is the largest organ in the human body, comprising ~40% of whole‐body mass in healthy adults.^1^ Crucial for many functions associated with whole‐body health beyond its most recognized role in locomotion, skeletal muscle also plays a fundamental role in energy homeostasis, oxygen consumption, energy metabolism, and substrate turnover and storage.^2^ A highly plastic tissue, skeletal muscle mass is maintained in health via a dynamic equilibrium between muscle protein synthesis (MPS) and muscle protein breakdown (MPB), with amino acid (AA) nutrition and contractile activity accepted as the most potent anabolic drivers.^3^ Pairing these drivers leads to increased MPS in response to nutrition^4^ and ultimately skeletal muscle hypertrophy [i.e., as is commonly seen with resistance exercise training (RET)]. Conversely, disuse or inactivity leads to reductions in MPS,^5^ ultimately leading to skeletal muscle atrophy. With skeletal muscle atrophy also a resultant impact of disease (i.e., cancer cachexia^6^), ageing (e.g., in sarcopenia^7^), and traumatic events (e.g., burns^8^ and sepsis^9^), when occurring as a result of decreased or absent contractile activity, it is often referred to as disuse muscle atrophy (DMA). As is seen in response to RET,^10^ a reproducible observation in relation to DMA is substantial inter‐individual heterogeneity^5^, highlighting the need for accessible methods to determine DMA across different cohorts and individuals.

Over the last five decades, there has been a step‐change in the methods available to quantify skeletal muscle size, including the introduction of magnetic resonance imaging (MRI^11^), computed tomography (CT^12^), and dual‐energy X‐ray absorptiometry (DXA^13^). Despite each of these methods being widely used, not only for assessment of muscle size, but also in clinical practice for a variety of diagnostic/prognostic endpoints (e.g., the assessment of brain lesions, tumour growth, and osteoporotic progression, respectively), they are each associated with expensive equipment, the need for highly trained operators/interpreters, and in the case of CT and DXA, ionizing radiation exposure.

In more recent years, ultrasound (U/S) has emerged as a potential additional tool for the assessment of muscle size in both young and older healthy cohorts,^14^, ^15^ and more recently in specific clinical cohorts including those with chronic obstructive pulmonary disease (COPD)^16^ or intensive care unit patients.^17^ Previous studies have reported a positive relationship between U/S‐derived measures of muscle thickness (MT_U/S_) and CT‐derived muscle size,^12^ DXA‐derived lean mass,^13^ and MRI (the gold standard for skeletal muscle size assessment)‐derived cross‐sectional area (CSA_MRI_) and muscle volume (VOL_MRI_).^18^

In addition to MT_U/S_, recent work has shown that muscle size measured as CSA by U/S (CSA_U/S_) also shows good agreement with MRI.^19^ This includes work by Stokes et al., who concluded, after a study of 10‐weeks RET to elicit hypertrophy and 2‐weeks immobilization of the contralateral limb, that CSA_U/S_ was a suitable alternative for measuring vastus lateralis (VL) changes in response to both increased and decreased muscle loading in young men.^20^ Similarly, Franchi and colleagues reported that RET‐induced hypertrophic changes in VL MT_U/S_ correlated with changes in VL CSA_MRI_, but not VOL_MRI._ ^14^ Beyond measures in the VL, Kositsky et al. showed that CSA_U/S_ can be used to reliably measure hamstring muscle and tendon size.^21^ Further, Sponbeck et al. showed a significant relationship between CSA_U/S_ and CSA_MRI_ across different posterior muscles of the lower leg.^19^

Despite this existent body of work, most previous studies that have assessed the utility of U/S (CSA_U/S_ and/or MT_U/S_) to measure muscle mass or size have reported on one muscle/muscle group only (e.g., VL^5^), with the upper portion of the leg (i.e., quadriceps and hamstrings) most common,^22^ likely given its functional importance in both athletic performance (e.g., jumping^23^) and activities of daily living (e.g., rising from a chair^24^). However, it has previously been shown that rates of DMA are not uniform across different muscles, even within a similar anatomical region (i.e., the lower leg^18^). In addition, the lower leg muscles have been shown to have significant functional importance in relation to gait and balance^25^ and as such, from a clinical perspective, falls prevention.^26^

Therefore, the aim of this study was to determine if U/S‐derived measures of MT_U/S_ or CSA_U/S_ could be used to accurately estimate changes in muscle size, as assessed by MRI, across different muscles of the lower leg known to have different profiles of atrophy susceptibility (i.e., tibialis anterior (TA) and medial gastrocnemius (MG)^18^).

## Methods

### Ethics approval and participants

This study was reviewed and approved by the University of Nottingham Faculty of Medicine and Health Sciences Research Ethics Committee (FMHS‐103‐1809) and registered online at ClinicalTrials.gov (NCT04199923). All procedures were conducted in accordance with the Declaration of Helsinki, and all participants provided written informed consent. All ethical guidelines for authorship and publishing in the *Journal of Cachexia, Sarcopenia and Muscle* were also followed.^27^

Ten recreationally active, young, healthy males (22 ± 4 years, 24.1 ± 2.3 kg/m^2^) participated in this study. Participants were screened by medical questionnaire, physical assessment, and resting electrocardiogram, with exclusions for cardiovascular, metabolic, and respiratory disorders, or other symptoms of ill‐health. Participants had clinically normal blood chemistry and pressure, were not prescribed any medication, undertook regular activities of daily living and recreation, but had not participated in any exercise training regime in the last 12 months.

### Experimental protocol

**Figure 1:**
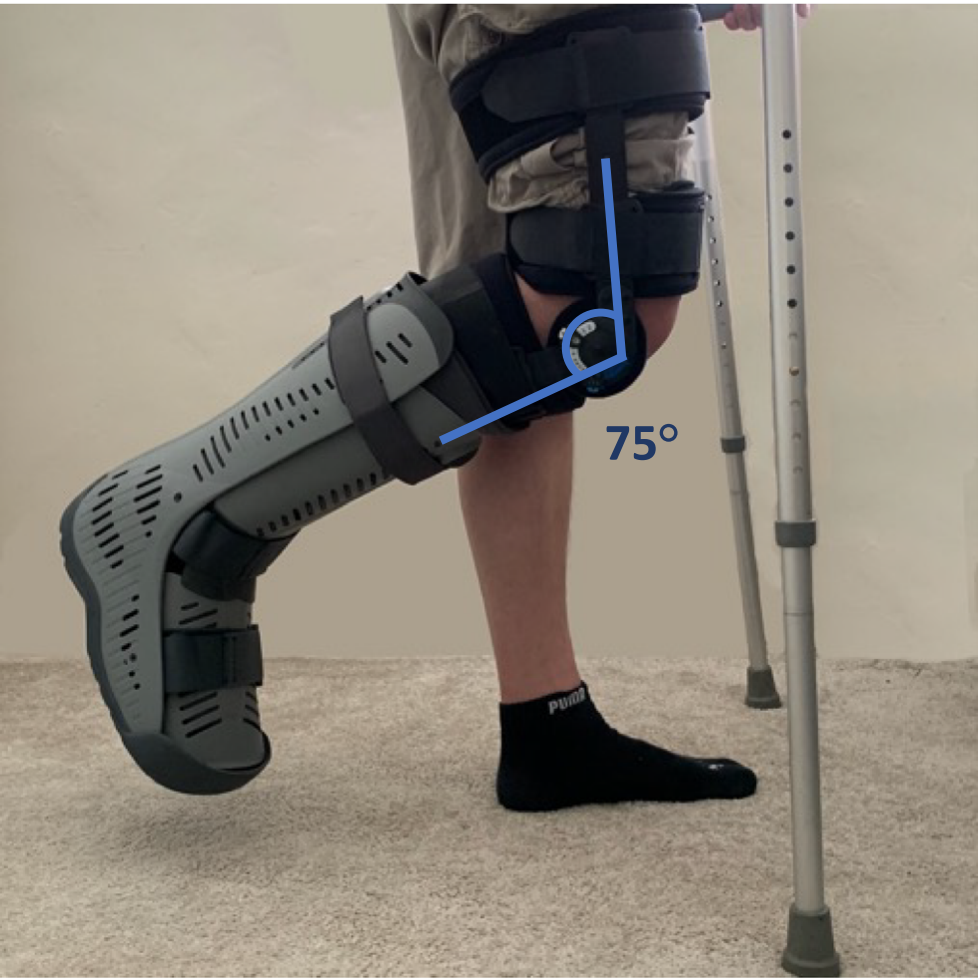
Representative unilateral lower leg immobilization using a leg brace and air‐boot, with supportive crutches for ambulation.

Each participant underwent 15 days of unilateral limb immobilization (ULI) using a hinged leg brace (Knee Post‐Op Cool) and air‐boot (Rebound Air Walker, both Ossur, Iceland), and ambulated on crutches (after training) throughout this period. The leg brace was fitted on the dominant leg over a compression sock around the thigh and lower leg, and fixed at 75° knee flexion to ensure no weight bearing could occur and allow sufficient ground clearance of the air‐boot (Figure 1). This leg was then also placed into an air‐boot with the ankle fixed in a neutral position to ensure no plantar or dorsi‐flexion (Figure 1). Signed ‘tamper tags’ were fitted to indicate if the brace or boot had been modified, which would have resulted in participant exclusion. No participants were excluded.

Prior to and after 15 days of immobilization, each participant visited the research unit for ultrasound and MRI analysis as described below. No adverse events were reported during this study.

### Magnetic resonance imaging

**Figure 2:**
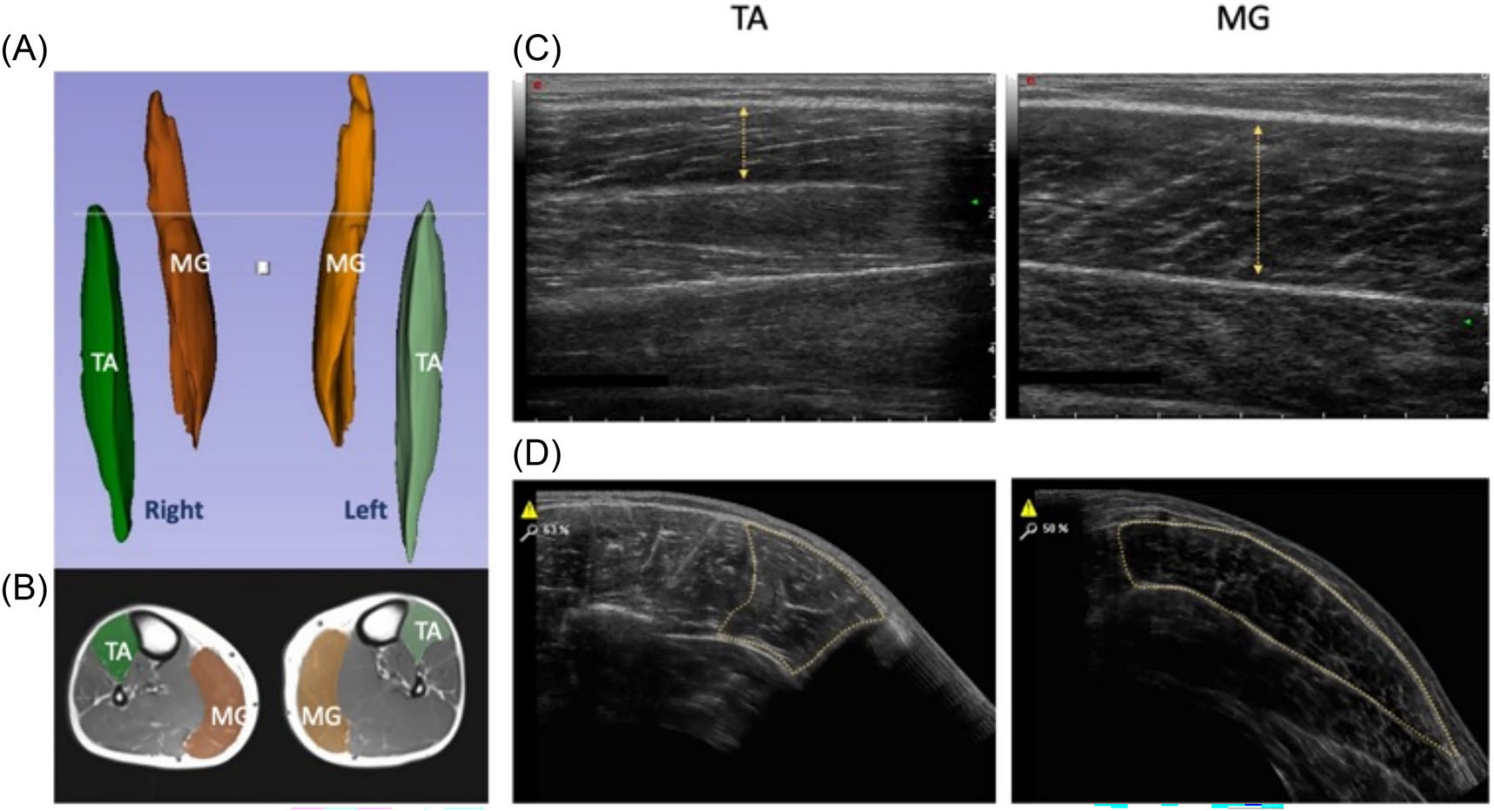
(A) MRI image of tibialis anterior (TA; green) and medial gastrocnemius (MG; orange) and representative 3D segmentation volume analysis. (B) MRI image with representative cross‐sectional area (CSA). Representative ultrasound images of TA (C) and MG (D) with muscle thickness and CSA analysis shown.

Participants were placed into the MRI scanner feet first, supine and instructed to relax for a minimum of 10‐min prior to scanning to normalize fluid shifts in the body. A 1.5T MRI system (Avanto, Siemens, Munich, Germany) was used to collect images of the leg from above the patella, facilitating collection of CSA_MRI_ and VOL_MRI_ measures from the TA and MG. An imaging matrix of 512 x 235 with a resolution of 835 x 835 μm was acquired with a slice thickness of 5 mm using a turbo spin echo sequence with an echo time set to the minimum value of 12 ms and a repetition time of 568 ms to optimize the trade‐off between imaging time and contrast for a proton density weighted image. A Siemens peripheral angiography coil was used to maximize the signal to noise ratio of resulting images. Scans were analysed by the same individual using Slicer (v4.10) software, with TA and MG individually segmented by pixel count every third slice before semi‐automatic filling between slices and confirming muscle boundaries to generate 3D muscle volumes (Figure 2A), with muscle cross‐sectional area measured at 50% of the length of the muscle, determined through the number of slices in each muscle (Figure 2B).

### Ultrasound imaging

After the MRI scan, ultrasound images were obtained with the participants leg extended and their ankle relaxed (~90°) as per the positioning for the MRI scans. All U/S scans were made with the probe resting on a gel layer without depressing the underlying skin.^28^ As previously described, TA and MG were scanned at 30% of their length on the mid‐sagittal line.^18^ TA was measured from the mid‐point of the patella on the anterior side of the leg to the fibula end, and MG from the inner knee crease to the fibula end. These anatomical landmarks were chosen to standardize scanning locations and consider variation in leg length between participants. For MT_U/S_ measures along with fibre length (Lf), images were captured using B‐mode ultrasonography (Mylab 70, Esaote Biomedica, Italy), with the transducer aligned in the fascicle plane (Figure 2C). Ultrasound Sarcopenia Index (USI)^29^ was calculated as the ratio between fibre length and muscle thickness (Lf/MT). CSA_U/S_ was measured using panoramic image acquisition^28^ in the axial‐plane at 30% of the muscle length (Figure 2D). Quantification of MT and CSA was then performed using ImageJ (Version 1.53) software, with MT_U/S_ determined as distance between the superficial and deep aponeuroses and an average across three images per muscle used for quantification. All ultrasound scans were performed and analysed in a blinded manner by the same individual.

### Statistical analysis

All analyses were performed using GraphPad Prism (v10.1.1). Correlative analysis was undertaken via Pearson's correlation, with *r* values stated. Columns depict mean ± SEM, with analysis via paired *t*‐tests within methodology. Bland–Altman analysis is reported as bias (SD of bias) and 95% limits of agreement. Significance was accepted as *P* < 0.05.

## Results

### Magnetic resonance imaging versus ultrasound for the assessment of muscle volume

**Figure 3:**
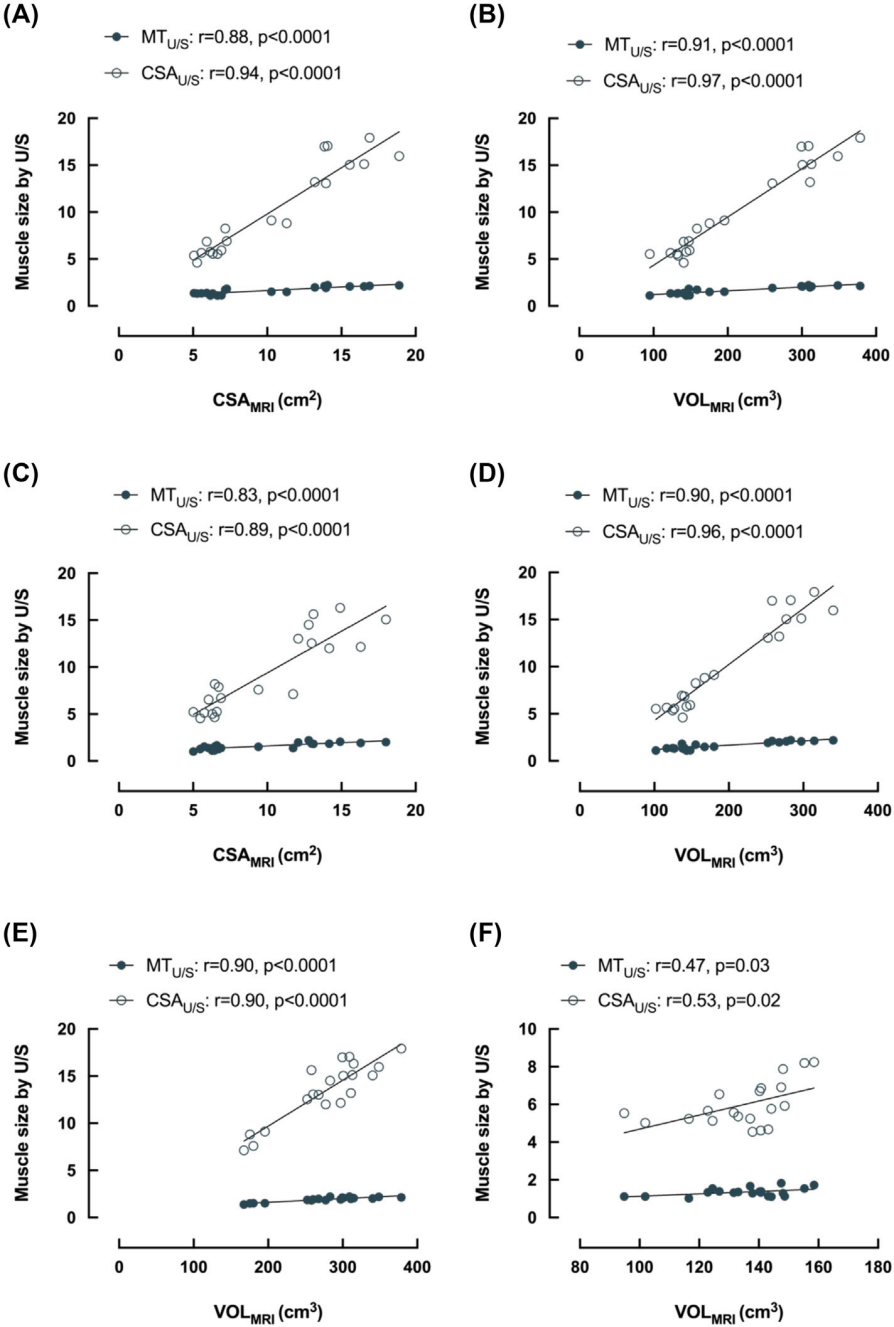
Relationships between muscle size (medial gastrocnemius (MG) and tibialis anterior (TA) combined) at baseline (A and B) and after 15‐day unilateral limb immobilization (C and D) in 10 individuals using magnetic resonance imaging (MRI) compared to ultrasound (U/S). MRI measures include cross‐sectional area (CSA) and muscle volume (VOL). U/S measures include CSA and muscle thickness (MT). Panels (E) and (F) show the relationship for VOL via MRI compared with both U/S measures for MG and TA, respectively. Analysis via Pearson's correlation. Significance accepted as *P* < 0.05.

Based on the entire data set of both muscles at baseline, there was a significant relationship between CSA_MRI_ and both U/S‐derived measures of MT_U/S_ (*r* = 0.88) and CSA_U/S_ (*r* = 0.94) (Figure 3A). There was also a significant relationship between VOL_MRI_ and both MT_U/S_ (*r* = 0.91) and CSA_U/S_ (*r* = 0.97) (Figure 3B), and between the two U/S measures (*r* = 0.93) and the two MRI measures (*r* = 0.96). The *P*‐value for each of these correlations was <0.0001.

After immobilization these relationships were maintained, with both CSA_MRI_ (Figure 3C) and VOL_MRI_ (Figure 3D) having a significant relationship with both MT_U/S_ (CSA_MRI_: *r* = 0.83; VOL_MRI_: *r* = 0.90) and CSA_U/S_ (CSA_MRI_: *r* = 0.90; VOL_MRI_: *r* = 0.96). The intra‐system relationships were also maintained (U/S: *r* = 0.86; MRI: *r* = 0.96). Unsurprisingly, when both timepoints were combined to offer an enhanced number of data sets for comparison, there remained a significant relationship between both CSA_MRI_ (MT_U/S_: *r* = 0.86; CSA_U/S_: *r* = 0.92) and VOL_MRI_ (MT_U/S_: *r* = 0.90; CSA_U/S_: *r* = 0.96) and each U/S measure. The *P*‐value for each of these correlations was <0.0001.

When both timepoints (pre‐ and post‐immobilization) were combined but the two muscles (TA and MG) were analysed separately, there was a significant relationship between CSA_MRI_ and both MT_U/S_ (*r* = 0.70, *P* = 0.0006) and CSA_U/S_ (*r* = 0.70, *P* = 0.0006) for MG, and stronger significant relationships between VOL_MRI_ and both MT_U/S_ (*r* = 0.90, *P* < 0.0001) and CSA_U/S_ (*r* = 0.90, *P* < 0.0001) (Figure 3E). For TA, the relationship between CSA_MRI_ and MT_U/S_ was non‐significant (*r* = 0.37, *P* = 0.10), and although the relationship with CSA_U/S_ was statistically significant, the relationship was weaker than for MG (*r* = 0.57, *P* = 0.009). Similarly, VOL_MRI_ measures of TA displayed significant but weaker correlations with both MT_U/S_ (*r* = 0.48, *P* = 0.03) and CSA_U/S_ (*r* = 0.53, *P* = 0.02) (Figure 3F).

Absolute values for both muscles via all methods pre‐ and post‐immobilization can be seen in Table S1.

### MRI versus ultrasound for the determination of atrophy susceptibility

Using VOL_MRI_ as the previously reported gold standard measure of muscle volume, there were clear differences in the rates of loss between muscles. The overall percentage loss across TA and MG combined was −5.21 ± 1.2%, with losses in each of these muscles −2.04 ± 1.31% and −8.40 ± 1.57%, respectively. Highlighting inter‐individual differences in DMA, overall changes ranged from −16.91 to 7.45% when both muscles were combined, with TA changes of −7.07 to 7.45% and MG changes of −16.91 to −2.37%.

**Figure 4:**
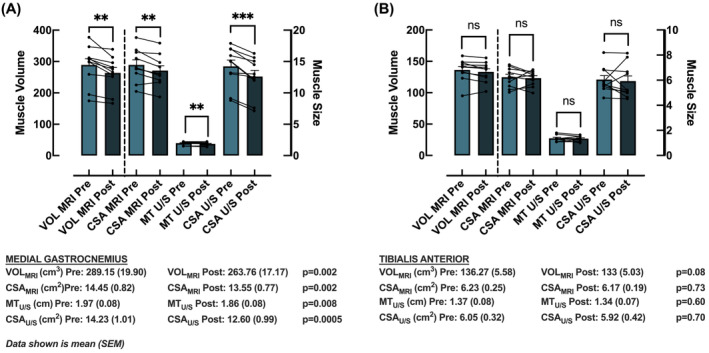
Skeletal muscle size of (A) medial gastrocnemius and (B) tibialis anterior at baseline (light) and after 15‐day unilateral limb immobilization (dark) in 10 individuals using magnetic resonance imaging (MRI) and ultrasound (U/S) methods. MRI measures include muscle volume (VOL) and cross‐sectional area (CSA). U/S measures include CSA and muscle thickness (MT). Values are mean ± SEM. Analysis via paired *t*‐tests within methodology. Significance accepted as *P* < 0.05. **P < 0.01; ***P < 0.001; ns = non‐significant.

Both U/S and MRI measures each detected significant DMA in the MG (CSA_MRI_, *P* = 0.02; VOL_MRI_, *P* = 0.002; MT_U/S_, *P* = 0.008; CSA_U/S_, *P* = 0.0005) (Figure 4A) but not the TA (CSA_MRI_, *P* = 0.74; VOL_MRI_, *P* = 0.08; MT_U/S_, *P* = 0.60; CSA_U/S_, *P* = 0.70) (Figure 4B).

Despite a significant relationship between both MRI measures and each U/S measure at baseline and after immobilization, and that each measure indicated atrophy resistance in the TA (compared to susceptibility in the MG), there was no significant relationship between either MRI measure with either U/S‐derived parameter for degree of DMA over the 15 days of immobilization. Initially analysed with both muscles combined and based on absolute values, this remained true when the two muscles were analysed separately, and when percentage change was used (Table 1).

**Table 1:** Relationships between disuse muscle atrophy measured using different imaging techniques after 15‐day unilateral limb immobilization in 10 individuals

Absolute change	MT_U/S_	CSA_U/S_
CSA_MRI_ ‐ Both muscles	*r* = 0.37, *P* = 0.11	*r* = 0.36, *P* = 0.12
CSA_MRI_ ‐ TA	*r* = 0.56, *P* = 0.09	*r* = 0.04, *P* = 0.92
CSA_MRI_ ‐ MG	*r* = −0.16, *P* = 0.66	*r* = −0.06, *P* = 0.88
VOL_MRI_ ‐ Both muscles	*r* = 0.24, *P* = 0.31	*r* = 0.40, *P* = 0.08
VOL_MRI_ ‐ TA	*r* = 0.22, *P* = 0.55	*r* = 0.22, *P* = 0.53
VOL_MRI_ ‐ MG	*r* = 0.02, *P* = 0.97	*r* = −0.08, *P* = 0.82
Percentage change		
CSA_MRI_ ‐ Both muscles	*r* = 0.44, *P* = 0.05	*r* = 0.09, *P* = 0.71
CSA_MRI_ ‐ TA	*r* = 0.54, *P* = 0.11	*r* = −0.009, *P* = 0.98
CSA_MRI_ ‐ MG	*r* = −0.39, *P* = 0.27	*r* = −0.35, *P* = 0.32
VOL_MRI_ ‐ Both muscles	*r* = 0.21, *P* = 0.39	*r* = 0.21, *P* = 0.35
VOL_MRI_ ‐ TA	*r* = 0.17, *P* = 0.65	*r* = 0.12, *P* = 0.75
VOL_MRI_ ‐ MG	*r* = −0.06, *P* = 0.87	*r* = −0.26, *P* = 0.47
Bland–Altman analysis		
CSA_MRI_ ‐ Both muscles	−0.41 (0.7), −1.8 to 0.96	0.39 (1.2), −1.9 to 2.7
CSA_MRI_ ‐ TA	−0.034 (0.50), −1.0 to 0.95	−0.06 (1.1), −2.2 to 2.3
CSA_MRI_ ‐ MG	−0.8 (0.68), −2.1 to 0.53	0.72 (1.2), − 1.6 to 3.1
VOL_MRI_ ‐ Both muscles	−14.18 (17.52), −48.52 to 20.15	−13.38 (17.1), −46.89 to 20.13
VOL_MRI_ ‐ TA	−3.1 (4.9), −13 to 6.5	−3 (4.8), −12 o 6.4
VOL_MRI_ ‐ MG	−25 (19), −62 to 11	−24 (19), −61 to 13

Abbreviations: CSA, cross‐sectional area; TA, tibialis anterior; MG, medial gastrocnemius; MRI, magnetic resonance imaging; VOL, volume; MT, muscle thickness; U/S, ultrasound. Analysis via Pearson's correlation and Bland–Altman analysis. Bland–Altman analysis shows bias (SD of bias) and 95% limits of agreement. Significance accepted as *P* < 0.05.

Recognizing the recent development of the USI as a tool to determine low muscle mass (albeit using the VL), we sought to determine if this method also had utility in determining the degree of DA in the TA and MG as muscles with different atrophy susceptibility profiles. The USI (where a higher value is associated with lower muscle mass) was able to identify the TA as atrophy resistant (pre‐immobilization: 5.10 ± 0.35 vs. post‐immobilization: 5.74 ± 0.36, *P* = 0.08) compared with the atrophy susceptible MG (1.90 ± 0.05 vs. 2.30 ± 0.18 post, *P* = 0.03), yet there was no relationship between the degree of DA in either muscle assessed by VOL_MRI_ (the gold standard) compared to the USI (*r* = 0.08, *P* = 0.75).

When the two muscles were grouped together Bland–Altman analysis suggests that compared to VOL_MRI_, both MT_U/S_ and CSA_U/S_ each appear to *underestimate* the degree of DMA. This was also true when comparing MT_U/S_ and CSA_U/S_ to CSA_MRI_ when analyzing the two muscles separately (Table 1).

### Baseline muscle size versus degree of disuse atrophy

Using the entire data set there was a significant relationship between baseline size and degree of DMA (absolute values) using both MRI methods (CSA_MRI_: *r* = −0.66, *P* = 0.002; VOL_MRI_: *r* = −0.78, *P* < 0.0001) and both U/S methods (CSA_U/S_: *r* = −0.61, *P* = 0.004; MT_U/S_: *r* = −0.48, *P* = 0.031), highlighting greater losses in those with larger baseline size. This remained true when DMA was considered as percentage change (data not shown).

However, when the muscles were analysed separately, there was no significant relationship between baseline muscle size and degree of absolute (TA, CSA_U/S_: *r* = −0.15, *P* = 0.68; MT_U/S_: *r* = −0.52, *P* = 0.13; MG, CSA_U/S_: *r* = −0.21, *P* = 0.57; MT_U/S_: *r* = −0.29, *P* = 0.42) or percentage change in either muscle via U/S. MRI methods did identify a significant relationship between baseline size and absolute DMA, although only via CSA_MRI_ for TA [*r* = −0.65, *P* = 0.041 (VOL_MRI_: *r* = −0.48, *P* = 0.16)] and VOL_MRI_ for MG [*r* = −0.58, *P* = 0.08 (CSA_MRI_: *r* = −0.37, *P* = 0.29)]. When DMA was presented as percentage change neither MRI method showed a significant relationship with baseline muscle size for either muscle.

### Intra‐system assessment of disuse muscle atrophy

Finally, when assessing DMA via the two different U/S measures (i.e., CSA_U/S_ vs. MT_U/S_) and the two different MRI measures (i.e., CSA_MRI_ vs. VOL_MRI_), there was a significant relationship for each only when the entire data set was used. There was no relationship between either intra‐system measures for TA when the muscles were analysed separately (Table 2), with a significant relationship only for the MRI measures for MG. Bland–Altman analysis suggests that compared to VOL_MRI_, CSA_MRI_ appears to underestimate the degree of DMA. Similarly, compared to MT_U/S,_ CSA_U/S_ also appears to underestimate muscle loss.

**Table 2:** Relationships between muscle atrophy measured using different imaging techniques on the same equipment after 15‐day unilateral limb immobilization in 10 individuals

Equipment	Correlation	Bland–Altman analysis
MRI	VOL_MRI_ vs. CSA_MRI_	Bias (SD), 95% limits of agreement
Both muscles	*r* = 0.68, *P* = 0.0009	−13.77 (17.06), −47.2 to 19.66
TA	*r* = 0.11, *P* = 0.77	−3.06 (4.9), −12.66 to 6.54
MG	*r* = 0.65, *P* = 0.04	−24.48 (18.3), −60.36 to 11.39
Ultrasound	MT_U/S_ vs. CSA_U/S_	
Both muscles	*r* = 0.48, *P* = 0.03	−0.81 (1.17), −3.11 to 1.5
TA	*r* = 0.48, *P* = 0.17	−0.09 (0.94), − 1.94 to 1.76
MG	*r* = 0.28, *P* = 0.43	−1.52 (0.95), −3.37 to 0.33

Analysis via Pearson's correlation and Bland–Altman analysis. Bland–Altman analysis shows bias (SD of bias) and 95% limits of agreement. Significance accepted as *P* < 0.05.

CSA, cross‐sectional area; MG, medial gastrocnemius; MRI, magnetic resonance imaging; MT, muscle thickness; TA, tibialis anterior; U/S, ultrasound; VOL, volume.

## Discussion

Assessment of changes in skeletal muscle size is an essential aspect of research related to DMA and clinical practice. Although MRI‐based measures are considered the gold standard, in recent years U/S has been promoted as a viable alternative for measuring muscle size, including in both hypertrophic^14^ and atrophic^30^ situations, due to its non‐invasive and generally accessible nature. However, the ability of U/S to assess DMA in muscles with differing degrees of atrophy susceptibility has yet to be determined. Here, we demonstrate that static measures of MT_U/S_ and CSA_U/S_ each strongly correlate with MRI‐derived measures of both CSA and VOL, before and after a period of immobilization. Moreover, when assessing individual muscles, these correlations were observed in muscles with both atrophy resistant (i.e., TA) and atrophy susceptible (i.e., MG) profiles. However, despite this, neither MT_U/S_ or CSA_U/S_ could resolve the degree of DMA in either muscle, or indeed when both muscles were combined.

Relatively short periods of disuse are known to rapidly induce DMA,^31^ with heterogenous rates of inter‐muscle atrophy observed as a result of, for example, prolonged bed‐rest.^32^ Moreover, we have previously characterized this apparent atrophy resistant versus atrophy susceptible (aRaS) paradigm in TA and MG muscles (respectively) in response to ULI,^18^ illustrating marked heterogeneity in inter‐muscle DMA even within a single anatomic region (i.e., the lower leg). Herein, we have demonstrated that two complementary U/S measures (i.e., CSA_U/S_ and MT_U/S_) are each viable methods to assess muscle size in concordance to the gold standard measure of VOL_MRI,_ both before and after immobilization. This was true for both atrophy susceptible (MG) and atrophy resistant (TA) muscle, albeit with a stronger correlation in MG compared to TA.

Although not focussed on inter‐muscle DMA, previous studies have shown MT_U/S_ be a reliable indicator of muscle size, including both before and after hypertrophic stimuli.^14^ However, similar to our findings for degree of atrophy, Franchi and colleagues reported that MT_U/S_ was not able to assess degree of hypertrophy in response to RET when compared to VOL_MRI_, postulating that this is likely due to regional changes across the muscle which are not accounted for by MT_U/S_ based on its assessment at a single region of the muscle only (i.e., mid‐point).^14^ Further, although Brook et al. reported DMA after 4 days of ULI using MT_U/S_, in this study MT_U/S_ was not compared to MRI, or indeed to any other imaging method. It did however report that both MT_U/S_ and DXA (lean mass) detected changes in the immobilized leg only, and that reductions in MT_U/S_ correlated with declines in MPS.^5^

Considering CSA_U/S,_ this method has previously been reported to accurately assess reductions in the size of atrophy susceptible muscles (i.e., MG and quadriceps) in response to sustained (i.e., 70 days) head‐down tilt bedrest,^28^ albeit with less favourable data (vs. CSA_MRI_) for MG compared to quadriceps. Conversely, although CSA_U/S_ measures of MG did show good agreement with both CSA_MRI_ and VOL_MRI_ at both time‐points (i.e., before and after ULI) in this study, it was not able to determine the magnitude of reductions in the size of MG (or TA) when compared to either MRI‐derived measure. This discrepancy may be due to sample size (with Scott and colleagues comparing ~700 images from 27 individuals) and/or the shorter duration of immobilization employed in our study.

When exploring intra‐system agreement, it is notable that only when both muscles were combined, were MT_U/S_ and CSA_U/S_ correlated. While this relationship is lost in individual muscles, this is likely due to statistical powering as post hoc analysis determines that correlations in MG atrophy between these two U/S methods would likely be observed with a minimum of 18 participants (α error probability = 0.05, power (1‐β) = 0.8, *r* = 0.28), a frequently reported sample size in clinical (e.g., critical care cohorts) and healthy volunteer cohorts.^33^ Further investigations utilizing U/S methods will help elucidate to determine the utility of ultrasound in clinical scenarios of muscle wasting.

Importantly, while both MRI and U/S methods were able to detect DMA in MG only, there is suggestion of a reduction in TA size via VOL_MRI_ only (*P* = 0.08). This is perhaps unsurprising given that geometric changes in different regions of a muscle are reflected only in VOL_MRI_ through successive measurement of CSA_MRI_ along the entire muscle length.^34^ Indeed, although measurement of CSA/MT_U/S_ at 30% of the muscle length (i.e., mid‐belly) has been shown to provide the greatest utility to detect changes in muscle size,^19^ it is likely that no measure at a single spatial location will truly reflect measures across the whole muscle.^35^ Specific to TA, it has been reported that only ~60% of variance in TA VOL may be attributable to MT.^36^ Despite this, although no significant loss in VOL_MRI_ was observed in TA in response to 15 days ULI, previous investigations have observed significant decreases in TA VOL_MRI_, but only following much longer periods of immobilization (i.e., 56 days bed rest in healthy individuals^32^). In addition, recent meta‐analysis shows dorsiflexor DMA of only −1.8% after 14 days immobilization^33^ across a range of bed‐rest studies each employing MRI_VOL._ As such, if the period of immobilization has been sufficient to elicit significant DMA in the TA, it is likely that this may only have been detected by MRI_VOL_.

The requirement for accessible methods to accurately determine muscle volume in different clinical contexts is essential to identify patients with low muscle volume, a major contributing factor in both mortality and morbidity,^37^, ^38^ and importantly aid in determining the clinical features of, for example cancer cachexia or sarcopenia.^39^ In addition, rates of DMA are also predictive of clinical outcomes, with these rates varying greatly between different clinical populations (e.g., ankle fracture vs. intensive care unit patients)^33^ and unsurprisingly being associated with severity of illness (i.e., single vs. multi organ failure).^30^ Effective and easy‐to‐access measurements of DMA may allow appropriate evaluation and intervention to preserve or reduce muscle loss in sub‐/clinical populations. For example, the recent development of an USI for the diagnosis of low muscle mass (i.e., sarcopenia) provides an inexpensive and clinically accessible tool centred on changes in muscle geometric proportions (i.e., MT and fibre length).^29^ Although in this study USI did not reflect varying degrees of DMA between different muscles, the full utility of this marker will clearly develop as the USI is validated in different (e.g.) clinical and ethnic populations, and perhaps different muscles. At present it only pertains to VL, likely due to both its fundamental role in locomotion and activities of daily living (e.g., rising from a chair), and ease of measurement. As significant heterogeneity in atrophy susceptibility between muscles is now clear, and lower leg muscles have been shown essential for gait and balance and therefore falls prevention,^26^ development of accessible tools such as the USI for application in other muscles may aid our understanding and mitigation of DMA. Further, in scenarios where panoramic image acquisition is not available (i.e., lack of appropriate software/hardware), the USI may be able to discern atrophy susceptibility through the acquisition of traditional static B‐mode ultrasound images.

In conclusion, this study demonstrates the utility of MT_U/S_ and CSA_U/S_ to assess size across muscles with divergent atrophy susceptibility profiles. Both U/S‐derived measures of muscle size were strongly correlated with those determined via MRI; however, neither could determine degree of DMA. Importantly U/S is already utilized within many clinical environments, as such, providing a relatively easy and quick assessment of muscle size in comparison to expensive and more restricted MRI. It is important to note that effective implementation of U/S requires consistent methodological employment (e.g., probe application) to ensure accurate assessment of muscle size. Future research should continue to investigate divergent responses between atrophy resistant/susceptible muscles and the patho/physiological importance of this paradigm, whilst also optimizing clinically accessible methods to assess muscle size in understudied muscles/muscle groups.

## Funding

This work was funded through a BBSRC grant (BB/R010358/1) and through the Medical Research Council (MRC), United Kingdom (grant no. MR/P021220/1) as part of the MRC‐Versus Arthritis Centre for Musculoskeletal Ageing Research awarded to the Universities of Nottingham and Birmingham. This work was also supported by the National Institute for Health Research, United Kingdom, Nottingham Biomedical Research Centre, and an MRC grant award (MR/X005240/1).

## Conflicts of Interest

All authors declare that this research was conducted in the absence of any commercial or financial relationships that could be construed as a potential conflict of interest.

## Supporting information


**Table S1.** Muscle ‘size’ measures of the medial gastrocnemius and tibialis anterior via magnetic resonance imaging (MRI: volume (VOL) and cross‐sectional area (CSA)) and ultrasound (U/S: muscle thickness (MT and CSA)).
