# Supplementary material for: Exploring the utility of ultrasound to assess disuse atrophy in different muscles of the lower leg
Source: J Cachexia Sarcopenia Muscle. 2024 Aug 26;15(6):2487–96. doi: 10.1002/jcsm.13583 (PMC11634512; doi:10.1002/jcsm.13583)
Supplement: Supplementary file 1 — Table S1. Muscle ‘size’ measures of the medial gastrocnemius and tibialis anterior via magnetic resonance imaging (MRI: volume (VOL) and cross‐sectional area (CSA)) and ultrasound (U/S: muscle thickness (MT and CSA)). [file JCSM-15-2487-s001.docx]

**Medial Gastrocnemius**

| **Measure** | **Pre (±SEM)** | **Post (±SEM)** | **P-value** |
| --- | --- | --- | --- |
| **VOL_MRI_ (cm^3^)** | 289.15 (19.90) | 263.76 (17.17) | p=0.002 |
| **CSA_MRI_** | 14.45 (0.82) | 13.55 (0.77) | p=0.002 |
| **MT_U/S_** | 1.97 (0.08) | 1.86 (0.08) | p=0.008 |
| **CSA_U/S_** | 14.23 (1.01) | 12.60 (0.99) | p=0.0005 |

**Tibialis Anterior**

| **Measure** | **Pre (±SEM)** | **Post (±SEM)** | **P-value** |
| --- | --- | --- | --- |
| **VOL_MRI_ (cm^3^)** | 136.27 (5.58) | 133 (5.03) | p=0.08 |
| **CSA_MRI_** | 6.23 (0.25) | 6.17 (0.19) | p=0.73 |
| **MT_U/S_** | 1.37 (0.08) | 1.34 (0.07) | p=0.60 |
| **CSA_U/S_** | 6.05 (0.32) | 5.92 (0.42) | p=0.70 |

**Table S1.** Muscle ‘size’ measures of the medial gastrocnemius and tibialis anterior via magnetic resonance imaging (MRI: volume (VOL) and cross-sectional area (CSA)) and ultrasound (U/S: muscle thickness (MT and CSA)).
